# The clinical trade-off of intraluminal versus extraluminal bronchial blockers in children: a retrospective analysis of respiratory mechanics and airway trauma during lung isolation preparation

**DOI:** 10.3389/fped.2026.1838411

**Published:** 2026-05-22

**Authors:** Zhiqing Gu, Change Zhu, Guoqing Wang, Lulu Ren, Sisi Wei, Rong Wei

**Affiliations:** Department of Anesthesiology, Shanghai Children's Hospital, Shanghai Jiao Tong University School of Medicine, Shanghai, China

**Keywords:** airway management, bronchial blocker, driving pressure, pediatric anesthesia, postoperative complications, respiratory mechanics

## Abstract

**Introduction:**

Optimal bronchial blocker (BB) placement for pediatric lung isolation presents a clinical dilemma. Intraluminal placement restricts the narrow endotracheal tube (ETT) lumen, whereas extraluminal placement risks upper airway trauma.

**Methods:**

This retrospective study compared adjusted respiratory mechanics and perioperative safety between intraluminal (Group I) and extraluminal (Group E) BB placement strategies in children. Primary outcomes included respiratory mechanics [peak airway pressure [Ppeak], airway resistance [Raw], and dynamic and static driving pressures [ΔPdyn, ΔPstat]] recorded during the lung isolation preparation phase prior to active one-lung ventilation (OLV). Bootstrapped multivariable linear regression was utilized to adjust for anatomical confounders, notably ETT size. Postoperative airway complications were assessed via video laryngoscopy.

**Results:**

A total of 68 children under 3 years of age were included in the final analysis, with 16 in Group I and 52 in Group E. The baseline characteristics were significant differences between the two groups (*p* < 0.05). After adjustment, Group I exhibited significantly worse respiratory mechanics, with profound elevations in Ppeak (*p* = 0.007), Raw (p < 0.001), ΔPdyn (*p* = 0.004), and ΔPstat (*p* = 0.014). The elevated ΔPstat strongly suggests occult auto-PEEP driven by severe expiratory flow limitation during this preparation phase. While neither group experienced intractable hypoxemia, procedure-related upper airway mucosal injuries exclusively occurred in Group E (*n* = 3).

**Discussion:**

In conclusion, a critical clinical trade-off is evident in pediatric lung isolation. While intraluminal bronchial blocker placement protects glottic structures from direct trauma, it profoundly increases airway resistance and the risk of auto-PEEP during the lung isolation preparation phase. Conversely, extraluminal placement significantly optimizes respiratory mechanics; however, our study unexpectedly revealed a distinct risk of adjacent upper airway mucosal injury exclusively with this technique. This novel finding underscores the necessity for anesthesiologists to strictly individualize the choice of bronchial blocker placement, carefully balancing the imperative for optimal respiratory mechanics against the potential for airway trauma based on patient airway anatomy and pulmonary compliance.

## 1 Introduction

One-lung ventilation (OLV) is a critical anesthetic technique frequently employed in pediatric thoracoscopic surgery to facilitate surgical exposure and minimize lung injury during thoracic procedures (1). Achieving effective lung isolation in infants and young children presents unique challenges due to their distinct airway anatomy, physiological vulnerabilities, and the limited availability of appropriately sized devices (2). Bronchial blockers (BBs) are increasingly preferred over double-lumen endotracheal tubes in this population due to their flexibility and ability to be used with standard endotracheal tubes (ETT), allowing for easier placement and maintenance in small airways (3).

However, the choice of BB placement technique—specifically whether the blocker is positioned intraluminally (within the ETT lumen) or extraluminally (alongside the ETT)—introduces a complex clinical trade-off. The intraluminal approach, while seemingly straightforward, can significantly reduce the functional cross-sectional area of the ETT, potentially increasing airway resistance and impairing respiratory mechanics (4). Conversely, the extraluminal approach preserves the ETT lumen but might pose a higher risk of upper airway trauma due to the presence of an additional device passing through the glottis (5). Despite the widespread use of BBs, a comprehensive understanding of the precise mechanical impact of these different placement strategies on pediatric respiratory mechanics, especially during the crucial lung isolation preparation phase, remains limited. Existing literature often focuses on the efficacy of lung collapse or complications during active OLV, rather than the immediate physiological consequences of the device's presence before surgery commences.

This study aimed to retrospectively evaluate and compare the impact of intraluminal versus extraluminal bronchial blocker placement on respiratory mechanics and safety outcomes during the lung isolation preparation phase in children undergoing thoracoscopic surgery. We hypothesized that the intraluminal placement would lead to increased airway resistance and dynamic hyperinflation (manifesting as elevated static driving pressure, ΔPstat) even during two-lung ventilation, while the extraluminal approach would preserve ideal respiratory mechanics. Additionally, we comprehensively reviewed perioperative records to identify any unanticipated safety outcomes associated with either placement technique.

## 2 Materials and methods

### 2.1 Study design and patient population

This retrospective cohort study was approved by the Institutional Review Board of Shanghai Children's Hospital (Approval No: 2022R115-E02), and the requirement for written informed consent was waived due to the retrospective nature of the analysis. We reviewed the medical records of children aged under 3 years who underwent elective thoracoscopic surgery requiring one-lung ventilation (OLV) between December 2023 and December 2025.

Patients were included if they required lung isolation using a bronchial blocker (BB) under pressure-controlled ventilation (PCV). Exclusion criteria were: (1) pre-existing severe cardiopulmonary diseases or significant airway anatomical anomalies; (2) American Society of Anesthesiologists (ASA) physical status IV or V; and (3) incomplete intraoperative respiratory mechanics data. (4) Height and/or weight outside the 25th to 75th percentiles for age. (5) absence of a preoperative chest CT scan report. (6) a history of upper respiratory tract infection within the preceding two weeks. Based on the BB placement technique, eligible patients were categorized into two groups: the intraluminal group (Group I) and the extraluminal group (Group E).

### 2.2 Anesthesia management and bronchial blocker placement

Upon arrival in the operating room, standard monitoring, including electrocardiography, non-invasive blood pressure, and pulse oximetry, was applied. According to routine institutional practice, general anesthesia was typically induced intravenously with sufentanil (2 μg/kg), glycopyrrolate (40 μg/kg), ciprofol (0.5 mg/kg), and rocuronium (0.6 mg/kg).

Following adequate muscle relaxation, tracheal intubation and bronchial blocker (BB) placement were performed by the attending anesthesiologist. A 5-Fr disposable endobronchial blocker tube (Tappa Medical Technology, China) was utilized in all cases. The decision to employ an intraluminal or extraluminal approach was not randomized; rather, it was based on the attending anesthesiologist's clinical judgment and specific surgical requirements. In cases categorized retrospectively into Group I, the BB had been advanced through the lumen of the endotracheal tube (ETT). In cases categorized into Group E, the extraluminal approach had been utilized, where the BB was placed parallel to the outer curve of the ETT. Correct positioning of the BB was routinely confirmed via fiberoptic bronchoscopy.

### 2.3 Routine ventilation protocol and data extraction

Following intubation and BB placement, mechanical ventilation was provided using an anesthesia machine (Carestation 650; GE Healthcare, Chicago, IL, USA) equipped with a dedicated respiratory airway module (E-sCAiOV, CARESCAPE™; GE Healthcare). The Pressure Controlled Ventilation-Volume Guaranteed (PCV-VG) mode was routinely initiated. As part of our institution's standard lung-protective ventilation strategy for pediatric thoracic surgery, the ventilator parameters during two-lung ventilation (TLV) were generally set as follows: initial target tidal volume (VT) at 10 ml/kg, positive end-expiratory pressure (PEEP) at 5 cmH_2_O, and respiratory rate (RR) at 20 breaths/min. The upper limit for the peak airway pressure alarm was typically set at 35 cmH_2_O, and the end-tidal carbon dioxide (ETCO_2_) was continuously monitored and maintained between 35 and 45 mmHg.

For this retrospective analysis, intraoperative data were extracted from the Anesthesia Information Management System (AIMS). To isolate the mechanical impact of the BB placement technique from confounding factors such as lateral decubitus positioning or surgical manipulation, respiratory mechanics variables were extracted at a specific and stable time point: after the completion of anesthetic induction and BB placement, with the patient in the supine position, prior to the inflation of the bronchial blocker balloon (i.e., during two-lung ventilation), and prior to the commencement of surgery.

At this designated time point, the primary respiratory mechanics variables were directly recorded from the GE respiratory monitor, including peak airway pressure (Ppeak), plateau pressure (Pplat), exhaled tidal volume (VTe), dynamic pulmonary compliance (Cdyn), airway resistance (Raw), and displayed PEEP. To comprehensively evaluate the respiratory mechanics and differentiate the resistive load from the elastic load, the following secondary parameters were calculated:

Dynamic driving pressure (ΔPdyn), representing the total mechanical load:

ΔPdyn=Ppeak−PEEPdisplayed

Static driving pressure (ΔPstat), reflecting the alveolar distending pressure:

ΔPstat=Pplat−PEEPdisplayed

Resistive pressure drop (ΔPres), quantifying the pressure dissipated to overcome airway resistance caused by the bronchial blocker:

ΔPres=Ppeak−Pplat

Additionally, to theoretically quantify the physical obstruction caused by the intraluminal BB, the reduction in cross-sectional area (ΔCSA) of the endotracheal tube was calculated based on the outer diameter of the 5-Fr BB and the inner diameter of the utilized ETT:

ΔCSA=(AreaBB/AreaETT)×100%

This theoretical variable was used to interpret the mechanical behavior of the respiratory system retrospectively.

### 2.4 Statistical analysis

Statistical analyses were performed using SPSS version 26.0 (IBM Corp., Armonk, NY, USA). Continuous variables were tested for normality using the Shapiro–Wilk test. Normally distributed data are presented as mean ± \pm ± standard deviation (SD) and were compared using the independent Student's *t*-test. Non-normally distributed data are expressed as median (interquartile range, IQR) and were compared using the Mann–Whitney *U* test. Categorical variables are presented as frequencies (percentages) and were analyzed using the Chi-square test or Fisher's exact test, as appropriate. A two-sided *P*-value of <0.05 was considered statistically significant.

## 3 Results

### 3.1 Patient enrollment and baseline characteristics

**Figure 1:**
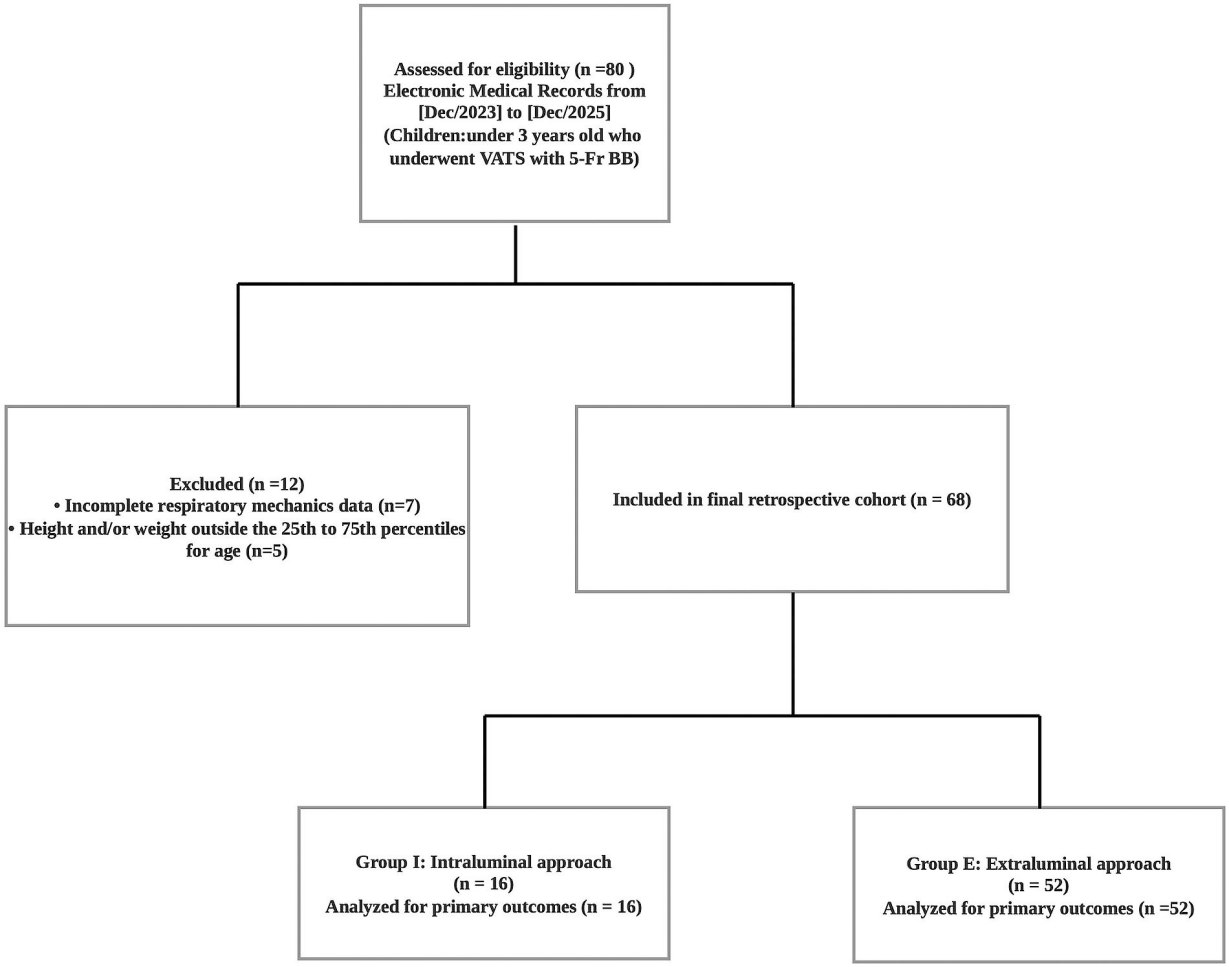
Study flowchart of patient enrollment, allocation, and follow-up for pediatric patients receiving intraluminal or extraluminal bronchial blockers.

Initially, a total of 80 children who underwent thoracic surgery with one-lung ventilation were assessed for eligibility. Among them, 12 patients were excluded: 7 due to incomplete perioperative medical records and 5 for having an age-adjusted height and/or weight outside the 25th to 75th percentiles. Consequently, 68 children were ultimately included in the final analysis. These patients were categorized into two cohorts based on the bronchial blocker placement technique: 16 patients in the intraluminal group (Group I) and 52 patients in the extraluminal group (Group E) (Figure 1).

Statistical analyses revealed significant differences across all baseline demographic and clinical variables between the two cohorts (Table 1). Patients in Group I were significantly older [median 10.50 [IQR: 6.00–22.50] months vs. 7.00 [IQR: 4.25–9.75] months; *p* = 0.014], heavier (10.34 ± 2.40 kg vs. 8.72 ± 1.57 kg; *p* = 0.02), and taller [median 74.00 [IQR: 69.25–83.75] cm vs. 69.00 [IQR: 66.00–74.75] cm; *p* = 0.025] compared to those in Group E. Regarding gender distribution, Group I had a higher proportion of females (68.8% vs. 28.8%; *p* = 0.007).There were no significant differences in mean arterial pressure, heart rate, pulse oxygen saturation, and end-tidal carbon dioxide (EtCO_2_) between the two groups (Supplementary Table S1).

**Table 1:** Baseline demographic and clinical characteristics of children undergoing thoracoscopic surgery, comparing intraluminal (Group I) and extraluminal (Group E) bronchial blocker placement.

Indicators	Group I (*n* = 16)	Group E (*n* = 52)	*p* value
Age (Months,m)	10.50 (6–22.5)	7.00 (4.25–9.75)	0.014*
Weight (Kilograms,kg)	10.34 ± 2.41	8.72 ± 1.57	0.02*
Height (Centimeters,cm)	74.00 (69.25–83.75)	69.00 (66.00–74.75)	0.025*
Sex (Male/Female)	5 (31.3%)/11 (68.8%)	37 (71.2%)/15 (28.8%)	0.007*
Endotracheal tube size (4/4.5/5.0)	9 (56.3%)/7 (43.8%)/0	47 (90.4%)/4 (7.7%)/1 (1.5%)	0.006*

Data are presented as mean ± SD, median (IQR), or *n* (%); *indicate statistical significance.

Furthermore, a significant discrepancy was observed in the selection of endotracheal tube (ETT) sizes (*p* = 0.006). Anesthesiologists tended to utilize larger ETTs in Group I, with 43.8% receiving a 4.5-mm ETT, whereas the vast majority (90.4%) of patients in Group E were successfully intubated with a 4.0-mm ETT. Given these baseline disparities, multivariable regression models were constructed to independently assess the impact of the placement techniques on respiratory mechanics while adjusting for these confounding factors.

### 3.2 Geometric assessment and unadjusted respiratory mechanics

Regarding the geometric characteristics of the airway, the median endotracheal tube (ETT) size was 4.0 (IQR, 4.0–4.5) mm in Group I and 4.0 (IQR, 4.0–4.0) mm in Group E. The intraluminal placement of the blocker in Group I resulted in a significant geometric reduction of the functional cross-sectional area, with a median ΔCSA of 17.43% (IQR, 13.77%–17.43%), whereas Group E maintained a fully patent lumen (ΔCSA = 0%) (*p* < 0.001).

**Figure 2:**
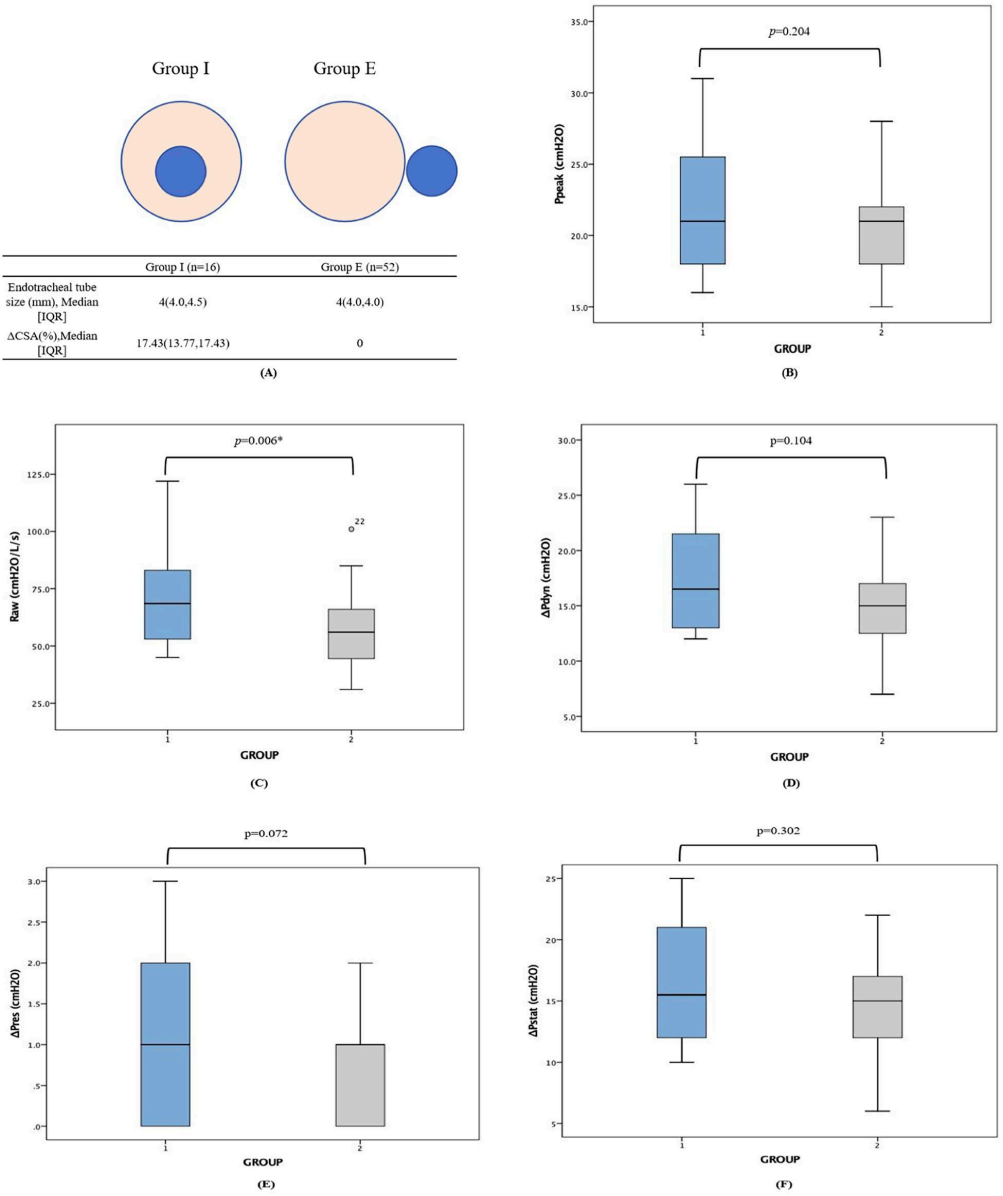
Airway geometry and unadjusted respiratory mechanics with the uninflated bronchial blocker *in situ*. **(A)** Schematic representation of the functional cross-sectional area reduction (ΔCSA). The intraluminal placement of the uninflated blocker shaft (Group I) caused a severe geometric reduction in the endotracheal tube lumen (median 17.43%) compared to the fully patent extraluminal approach (Group E, 0% reduction). **(B–E)** Box plots illustrating the unadjusted comparisons of peak airway pressure [Ppeak, **(B)**], airway resistance [Raw, **(C)**], dynamic driving pressure [ΔPdyn, **(D)**], and resistive driving pressure [Pres, **(E)**] measured during two-lung ventilation immediately after blocker placement. In this unadjusted univariate analysis, only Raw was significantly higher in Group I (*p* = 0.006). Other pressure parameters showed no superficial statistical differences between the two groups (*p* > 0.05), a phenomenon inherently confounded by the baseline demographic disparities (larger body size and ETT diameter in Group I). The central horizontal lines represent the median, the boxes span the interquartile range (IQR), and the whiskers indicate the minimum and maximum values. Statistical comparisons were performed using the independent-samples *t*-test for normally distributed data and the Mann–Whitney *U* test for non-normally distributed data. Group I, intraluminal group; Group E, extraluminal group; ETT, endotracheal tube. *Indicates statistical significance (*p* < 0.05).

During lung isolation preparation, unadjusted univariate analysis revealed that airway resistance (Raw) (70.63 ± 21.38 vs. 57.25 ± 14.50) was significantly higher in Group I compared to Group E (*p* = 0.006). However, despite the severe luminal compromise in Group I, no statistically significant differences were observed between the two groups in terms of peak airway pressure (Ppeak), dynamic driving pressure (ΔPdyn), resistive driving pressure (ΔPres), and static driving pressure (ΔPstat) (all *p* > 0.05) (Figure 2).

### 3.3 Multivariable analysis of respiratory mechanics

To eliminate the confounding effects of baseline demographic and anatomical disparities (including age, weight, height, and ETT size) observed between the two groups, multivariable regression analyses were performed (Table 2). Following adjustment for these covariates, the independent impact of the intraluminal blocker placement on respiratory mechanics was unmasked.

**Table 2:** Multivariable linear regression analysis of intraoperative respiratory mechanics in pediatric patients, adjusting for baseline demographic and anatomical disparities (age, weight, height, and ETT size) between group I and group E.

Variable	Adjusted Coefficient (*β*) for Group I vs. E	95% Confidence Interval	*P* value
Ppeak (cmH2O)	2.73	0.79,4.67	0.007*
Raw (cmH2O/L/s)	20.62	11.76,29.47	0.000*
ΔPdyn (cmH2O)*	3.88	1.30,6.74	0.004*
ΔPres (cmH2O)*	0.32	−0.11,0.75	0.176
ΔPstat (cmH2O)*	3.57	0.84,6.60	0.014*

*Indicate statistical significance; ETT, endotracheal tube; Ppeak, peak airway pressure; Raw, airway resistance; ΔPdyn, dynamic driving pressure; ΔPres, resistive pressure drop; ΔPstat, static driving pressure.

The multivariable models revealed that Group I was independently associated with significantly higher peak airway pressure (Ppeak, *p* = 0.007) and dynamic driving pressure (ΔPdyn, *p* = 0.004) compared to Group E. Moreover, the robust statistical significance of airway resistance was maintained, indicating severely elevated resistance in Group I (Raw, *p* < 0.001).

Interestingly, the adjusted analysis also demonstrated a significantly higher static driving pressure (ΔPstat) in Group I (*p* = 0.014). Concurrently, the independent association between Group I and resistive driving pressure (ΔPres) did not reach statistical significance (*p* = 0.176), potentially due to the proportional elevation of both peak and plateau pressures secondary to blocker-induced flow limitation.

### 3.4 Safety outcomes and adverse events

**Figure 3:**
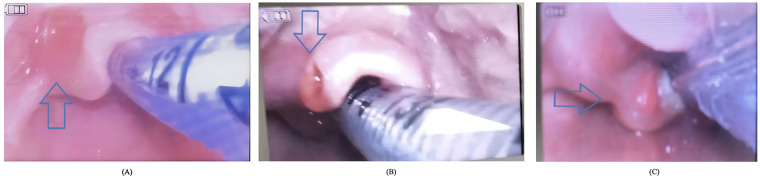
Upper airway complications associated with extraluminal bronchial blocker placement in children under 3 years old. Representative images obtained via video laryngoscopy during postoperative blocker extraction in Group E. **(A)** Localized epiglottic edema adjacent to the blocker shaft (blue arrow). **(B)** Mucosal abrasion on the epiglottis contiguous to the blocker (blue arrowhead). **(C)** Unilateral arytenoid swelling and edema on the side of blocker placement (blue arrow). No such complications were observed in Group 1. Group E, extraluminal group; Group I, intraluminal group.

Regarding perioperative safety, no patients in either group experienced intractable hypoxemia requiring premature termination of lung isolation. However, procedure-related upper airway complications were exclusively observed in the extraluminal group (Group E, *n* = 3, 5.8%). These minor traumatic events were identified under direct vision using video laryngoscopy during the extraction of the bronchial blocker at the end of surgery (Figure 3). Specifically, the documented injuries included: localized epiglottic edema adjacent to the blocker shaft (*n* = 1), a mucosal abrasion on the epiglottis contiguous to the blocker (*n* = 1), and unilateral arytenoid edema on the ipsilateral side of the blocker placement (*n* = 1). No upper airway traumatic events were reported in the intraluminal group (Group I).

## 4 Discussion

The present study addresses a critical clinical dilemma during the preparation for pediatric lung isolation: optimizing respiratory mechanics while minimizing airway trauma when deploying a bronchial blocker (BB). Our principal findings demonstrate a fascinating physiological paradox. Initially, unadjusted univariate analysis suggested that intraluminal BB placement (Group I) caused no significant deterioration in airway pressures compared to the extraluminal approach (Group E), except for an expected rise in airway resistance (Figure 2). However, multivariable regression analysis unmasked the truth: after adjusting for the larger baseline endotracheal tube sizes and greater physical stature in Group I, the intraluminal placement was independently associated with profound deteriorations in peak airway pressure (Ppeak), dynamic driving pressure (ΔPdyn), and static driving pressure (ΔPstat) (Table 2). Conversely, while Group E demonstrated superior respiratory mechanics, it was exclusively associated with procedure-related upper airway mucosal injuries (Figure 3).

The masking effect observed in our preliminary data underscores the nonlinear relationship between airway radius and resistance. According to the Hagen–Poiseuille equation, resistance is inversely proportional to the fourth power of the radius (6). In Group I, the intraluminal blocker caused a significant geometric compromise, reducing the cross-sectional area (ΔCSA) by a median of 17.43%. This geometric penalty and its subsequent mechanical burden are highly consistent with the pediatric lung model studies by Hsia et al. (7), who demonstrated that the insertion of a device (such as a flexible bronchoscope) into an ETT severely compromises the functional lumen, leading to exponential increases in peak airway pressure. Their findings corroborate our observation that even marginal luminal compromises in pediatric micro-airways provoke disproportionate mechanical deterioration. In a clinical scenario without adjustment, the use of larger ETTs (e.g., 4.5 mm) in Group I offset this luminal loss, creating a “false equivalence” in driving pressures when compared to smaller children with intact lumens in Group E. By employing multivariable regression, we successfully eliminated these baseline confounding factors, revealing the true mechanical burden of the intraluminal shaft, which drastically elevated Ppeak, ΔPdyn, and Raw.

A particularly novel and counterintuitive finding in our adjusted model was the significant elevation of ΔPstat in Group I (*p* = 0.014), alongside the lack of a statistically significant divergence in resistive driving pressure ΔPres, *p* = 0.176). Traditionally, ΔPstat reflects the intrinsic compliance of the lung parenchyma and chest wall, which should remain theoretically unaffected by a rigid device in the trachea (8). We postulate that this phenomenon is entirely driven by severe expiratory flow limitation. The substantial reduction in ΔCSA not only impedes inspiratory flow but also severely prolongs the expiratory time constant. Under standard ventilator settings, this inadequate expiratory time inevitably leads to gas trapping and the generation of intrinsic positive end-expiratory pressure (auto-PEEP) (9). This pathophysiological mechanism is strongly supported by Lawson et al. (10), who revealed that an intraluminal shaft dramatically curtails expiratory flow, generating significant auto-PEEP and posing a high risk of occult barotrauma. Consistent with their findings, because our measurement of ΔPstat relies on the subtraction of set extrinsic PEEP rather than total PEEP, the undetected auto-PEEP artificially inflates the plateau pressure (11). Therefore, the elevated ΔPstat in Group I does not imply stiffening lungs, but rather serves as a surrogate marker for dynamic hyperinflation—a critical warning sign for pediatric anesthesiologists that intraluminal placement may cause hidden barotrauma. It is striking that these severe mechanical deteriorations were observed even before the initiation of single-lung ventilation, purely as a result of the intraluminal device occupying the restricted ETT cross-section.

In stark contrast, the extraluminal technique (Group E) preserves the entirety of the ETT lumen (ΔCSA = 0%), thereby mitigating expiratory flow resistance and completely avoiding auto-PEEP. Importantly, the potential for procedure-related upper airway injury secondary to extraluminal placement was an unexpected clinical finding that we did not initially anticipate. Our safety outcomes indicate that the mechanical superiority of the extraluminal approach comes at an anatomical cost. The physical presence of the blocker shaft traversing the glottis adjacent to the ETT exerts continuous mechanical pressure on vulnerable pediatric mucosal surfaces (9). This was corroborated by our video laryngoscopic findings, which documented three cases of upper airway trauma—including localized epiglottic edema, mucosal abrasions, and arytenoid swelling—exclusively in Group E. Previous research, including a study from our institution by Yan et al. (12), has established the overall clinical safety of extraluminal placement in infants, demonstrating no increased incidence of postoperative hoarseness. Building upon these foundational clinical observations, our current study aimed to provide a more granular anatomical assessment. We revealed that while gross clinical symptoms (e.g., hoarseness) may be absent, direct visual inspection can detect a 5.8% incidence of subclinical, occult mucosal injuries. This suggests that while the extraluminal approach is clinically well-tolerated, clinicians must remain vigilant about the physical friction exerted by the shaft. These injuries, though minor in our cohort, theoretically increase the risk of postoperative stridor and airway compromise in infants (11).

Thus, the choice between intraluminal and extraluminal placement represents a classic clinical trade-off that necessitates a highly individualized approach. The intraluminal approach protects the glottic structures but severely penalizes respiratory mechanics, predisposing the child to auto-PEEP and barotrauma. The extraluminal approach optimizes ventilation and gas exchange but increases the risk of direct vocal cord and epiglottic trauma. From a practical clinical perspective, for children with poor pulmonary compliance or those requiring stringent lung-protective ventilation, the extraluminal approach is highly advantageous, provided that rigorous video-laryngoscopic guidance is employed to minimize trauma. Conversely, in patients with anticipated difficult airways or fragile mucosal anatomy, the conventional intraluminal approach remains the safer alternative, albeit requiring meticulous ventilator adjustments to counteract occult auto-PEEP (13).

## 5 Limitations

Our study has several limitations. First, its retrospective, observational nature precludes the establishment of definitive causality, despite our robust multivariable adjustments. Second, the presence of occult auto-PEEP was deduced from the elevation in ΔPstat and mechanical principles, rather than being directly quantified using an end-expiratory hold maneuver; future prospective studies should incorporate direct measurements of intrinsic PEEP. Third, because the upper airway trauma was an unanticipated finding, our study was not prospectively designed to grade these mucosal injuries systematically or correlate them with detailed preoperative anatomical assessments (e.g., Cormack-Lehane classifications). Finally, as previously emphasized, our respiratory mechanics data were collected with the bronchial blocker *in situ* but prior to the actual execution of one-lung ventilation. While this approach cleanly isolates the mechanical impact of the placement strategy itself without the confounding factors of surgical retraction or lung collapse, further research is required to evaluate how these baseline mechanical compromises evolve dynamically during prolonged pediatric one-lung ventilation.

## 6 Conclusion

In summary, while the intraluminal placement of a bronchial blocker protects pediatric upper airway structures from mechanical trauma, it severely compromises the functional airway lumen, independently driving up airway resistance, peak pressures, and the risk of dynamic hyperinflation. Extraluminal placement provides ideal respiratory mechanics but carries a distinct and unexpected risk of adjacent mucosal injury. Rather than adopting a universal protocol, anesthesiologists must strictly individualize the choice of BB placement strategy, carefully balancing the need for optimal lung-protective ventilation against the patient's baseline airway anatomy and the anticipated duration of one-lung ventilation.

## Data Availability

The original contributions presented in the study are included in the article/Supplementary Material, further inquiries can be directed to the corresponding author.
