# Supplementary material for: The clinical trade-off of intraluminal versus extraluminal bronchial blockers in children: a retrospective analysis of respiratory mechanics and airway trauma during lung isolation preparation
Source: Front Pediatr. 2026 May 22;14:1838411. doi: 10.3389/fped.2026.1838411 (PMC13236636; doi:10.3389/fped.2026.1838411)
Supplement: Supplementary file 1 [file Table1.docx]

Supplementary Material

# Supplementary Table

**Supplementary** **Table 1:**Comparison of intraoperative vital signs between pediatric patients receiving intraluminal (Group I) and extraluminal (Group E) bronchial blocker placement.

| Indicators | Group I (n=16) | Group E (n=52) | *p* value |
| --- | --- | --- | --- |
| Heart Rate (HR,bpm) | 139.50 (136.25,142.75) | 140.50 (128.00,153.00) | 0.734 |
| Blood Pressure (mean,mmHg) | 65.50 (63.25,66.75) | 68.00 (63.00,71.00) | 0.132 |
| SpO_2_ (%) | 99.00 (99.00,100.00) | 100.00 (99.00,100.00) | 0.159 |
| EtCO_2_ (mmHg) | 39.00 (35.35,40.75) | 38.00 (34.00,44.00) | 0.673 |

SpO2 ,pulse oxygen saturation;EtCO2,
